# Glycoscience data content in the NCBI Glycans and PubChem

**DOI:** 10.1007/s00216-024-05459-7

**Published:** 2024-08-12

**Authors:** Sunghwan Kim, Jian Zhang, Tiejun Cheng, Qingliang Li, Evan E. Bolton

**Affiliations:** National Center for Biotechnology Information, National Library of Medicine, National Institutes of Health, Bethesda, MD 20894 USA

**Keywords:** Glycan, PubChem, GlyTouCan, GlyCosmos, GlyGen, PUG-View

## Abstract

Studying﻿ glycans and their functions in the body aids in the understanding of disease mechanisms and developing new treatments. This necessitates resources that provide comprehensive glycan data integrated with relevant information from other scientific fields such as genomics, genetics, proteomics, metabolomics, and chemistry. The present paper describes two resources at the U.S. National Center for Biotechnology Information (NCBI), the NCBI Glycans and PubChem, which provide glycan-related information useful for the glycoscience research community. The NCBI Glycans (https://www.ncbi.nlm.nih.gov/glycans/) is a dedicated website for glycobiology data content at NCBI and provides quick access to glycan-related information scattered across multiple NCBI databases as well as other information resources external to NCBI. Importantly, the NCBI Glycans hosts the official web page for the symbol nomenclature for glycans (SNFG), which is the standard graphical representation of glycan structures recommended for scientific publication. On the other hand, PubChem (https://pubchem.ncbi.nlm.nih.gov) is a research-focused, large-scale public chemical database, containing a substantial number of glycan-containing records and is integrated with important glycoscience resources like GlyTouCan, GlyCosmos, and GlyGen. PubChem organizes glycan-related information within multiple data collections (i.e., Substance, Compound, Protein, Gene, Pathway, and Taxonomy) and provides various tools and services that allow users to access them both interactively through a web browser and programmatically through a REST-ful interface, including PUG-View. The NCBI Glycans and PubChem highlight glycan-related data and improve their accessibility, helping scientists exploit these data in their research.

**Graphical Abstract:**

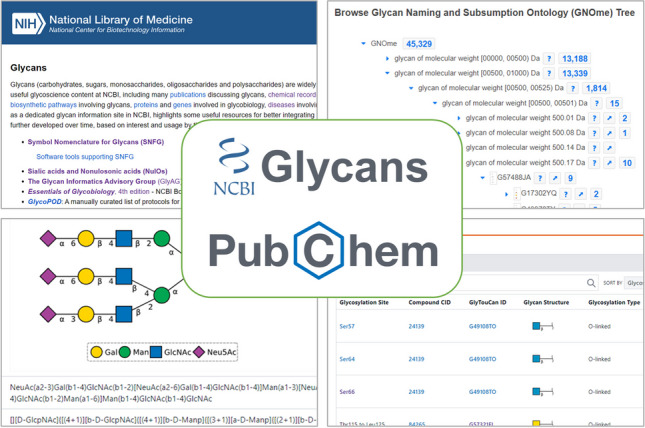

**Supplementary Information:**

The online version contains supplementary material available at 10.1007/s00216-024-05459-7.

## Introduction

Glycans play crucial roles in various biological processes [1–5]. As major components of the cell surface, they are involved in cell-cell communications and signaling events. Recognized by microbes (e.g., viruses and bacteria), glycans influence the host-pathogen interaction in infectious disease at various stages from initial colonization to tissue spread and inflammation [6]. In addition, defects in glycan synthesis, metabolism, and recognition are associated with various diseases [7], such as cancer [8–10] and congenital disorders of glycosylation (CDGs) [11, 12]. Therefore, studying glycans and their roles in the body helps in understanding disease mechanisms and developing novel therapeutics. This requires resources that provide comprehensive glycan data to be integrated with relevant data in other scientific areas such as genomics, genetics, proteomics, metabolomics, and chemistry.

Since the development of CarbBank [13] in the late 1980s, many glycan-related information resources and coordinated efforts have emerged, including Glycome Atlas [14, 15], the Carbohydrate Structure Databases [16], the carbohydrate-active enzyme (CAZy) database [17], Glyco Epitope [18], Total Glycome Database [19], KEGG Glycan [20], GlyTouCan [21, 22], GlyComb [23], GlyPOST [24], UniCarb-DR [25], GlyGen [26], Glycomics@ExPASy [27], GlyCosmos Portal [28], GlySpace Alliance [29, 30], and many others. To help users more efficiently use glycan data scattered across many different resources, there have been collaborative efforts to promote data exchange and integration among glycoscience data resources [28–30]. An example is the GlyCosmos Portal [28], which integrates glycan data repositories, including GlyTouCan (for glycan structures) [21, 22], Glycomb (for glyconjugate data) [23], GlyPOST (for glycomics mass-spectrometry (MS) raw data) [24], and UniCarb-DR (for glycomics MS peak lists) [25]. The GlyCosmos Portal also integrates glycan data from multiple databases and provides quick access to multi-omics data for glycans. Another example of collaborative efforts in glycoscience is the Glyspace Alliance [29, 30], a tri-continent alliance formed by the teams responsible for three major glycoscience data integration projects: GlyGen (in the USA) [26], Glycomics@ExPASy (in Switzerland) [27], and the GlyCosmos Portal (in Japan) [28]. The GlySpace Alliance aims to provide high-quality glycan-related data, by freely sharing relevant information among its participating members.

The U.S. National Center for Biotechnology Information (NCBI) manages two glycan-related information resources: NCBI Glycans and PubChem [31–34]. The NCBI Glycans (https://www.ncbi.nlm.nih.gov/glycans/) website, a dedicated glycan information portal at NCBI, provides quick access to glycoscience data content scattered across multiple NCBI databases as well as other databases external to NCBI. PubChem [31–34] is a large-scale public chemical database and has a substantial amount of glycan-related data, including curated information such as experimental bioactivities of glycans. The present paper describes an overview of these two resources, including data sources and organization as well as tools and services useful to researchers interested in glycoscience content. In addition, it also explains the current limitations of the two resources and the ongoing efforts to address them.

## Material and methods

### Data sources

PubChem data are organized into multiple data collections [35], including Substance, Compound, BioAssay, Gene, Protein, Pathway, Cell Line, Taxonomy, and Patent. While the Substance and BioAssay collections serve as archives that keep chemical information voluntarily submitted by data sources, the other collections serve as knowledgebases that provide users with easy access to organized high-quality information about PubChem records.

Considering PubChem’s dual role as an archive and as a knowledgebase, its data sources can be broadly classified into two groups: (1) archival data sources, which voluntarily submit their data to PubChem Substance and BioAssay, and (2) annotation sources, from which the PubChem team collects authoritative and curated chemical information to annotate PubChem records. Some sources may belong to both groups.

PubChem has almost one thousand data sources (996 sources as of June 7, 2024). Some of them are popular glycoscience information resources, including GlyTouCan [21, 22], GlyGen [26], GlyCosmos Glycoscience Portal [28], and GlycoNAVI. These resources are used to annotate various types of PubChem records (such as compounds, proteins, genes, pathways, and organisms).

It is noteworthy that GlyTouCan is also an archival data source within PubChem, meaning that its glycan records can be found in PubChem Substance. Currently, there are about 220 thousand substances provided by GlyTouCan and about 40% (84 thousand substances) of them are associated with PubChem Compound records.

### Data integration

For the 220 thousand substances from GlyTouCan, their Web3.0 Unique Representation of Carbohydrate Structure (WURCS) strings [36, 37] were used to generate molecular structures in a structure-data file (SDF) format, using MolWURCS (https://gitlab.com/glycoinfo/molwurcs), which is designed to interconvert between WURCS and several other file formats used in molecular modeling, computational chemistry, and related area. The generated structures were processed through chemical structure standardization [38]. If the standardized structure of a glycan already exists in PubChem Compound, the association between the substance and the compound was generated. If the standardized structure did not exist in PubChem Compound, a new compound record was created before the association was generated.

Glycan-related data used to annotate PubChem records were obtained from annotation data sources, including GlyGen, GlyTouCan, GlyCosmos, and GlycoNAVI (see Table 1). These data were mapped to corresponding records in PubChem. The identifiers used for the data mapping were the GlyTouCan IDs for compounds and the NCBI Gene IDs and protein accessions for genes and proteins, respectively. For taxa, the NCBI Taxonomy IDs were used. For pathways, the Reactome IDs were used.

**Table 1:** Glycan-related annotations in PubChem

Record type	Annotations	Annotation sources
Compound	Biological Line Notations	GlyTouCan
	GlyTouCan Accession	GlyTouCan, GlyCosmos, GlycoNAVI
	GlyGen Classification	GlyGen
	GlyGen Motifs	GlyGen
	Monosaccharide Composition	GlyGen
	Permethylated Mass	GlyGen
	Chemical-Target Interactions	GlyCosmos
	GlyCosmos Monoisotopic Mass	GlyCosmos
	GlyCosmos Species	GlyCosmos
	GlyCosmos Subsumption	GlyCosmos
	GlyCosmos Tissue/Organ	GlyCosmos
	Record Description	GlyCosmos
	Taxonomy	GlyCosmos
Protein	GlyGen Protein	GlyGen
	Glycosylation	GlyGen
	GlyCosmos Protein	GlyGen, GlyCosmos
Gene	Chemical-Gene Interactions	GlyCosmos
	Gene-Disease Associations	GlyCosmos
	GlyCosmos Gene	GlyCosmos
Pathway	Glycogenes	GlyCosmos
	Glycopathways	GlyCosmos
	Glycoproteins	GlyCosmos
	Glycans	GlyCosmos
Taxonomy	Glycans	GlyCosmos

### Data presentation

As mentioned previously, PubChem has multiple data collections. Each record in these collections has a Summary page, which is a dedicated web page that shows all information for that record available in PubChem. The glycan-related annotations for a PubChem record are presented in the appropriate section of the Summary page for that record.

## Results and discussion

### NCBI Glycans

The NCBI Glycans (https://www.ncbi.nlm.nih.gov/glycans/), launched in September 2017, serves as a central gateway for glycoscience researchers by providing quick access to a wealth of information scattered across multiple information resources. It provides links to two popular online books, “Essentials of Glycobiology” [39] and “Glycoscience Protocols (GlycoPODv2)” [40], freely available at the NCBI Bookshelf. The “Essentials of Glycobiology” is an online textbook for upper-undergraduate and graduate students majoring in life sciences and biomedicine, where it provides a basic overview of glycobiology and covers a wide range of topics, from biology and medicine to chemistry, bioenergy, and material science. The “Glycoscience Protocols” contains free online protocols about experiments commonly performed in glycoscience research. These protocols were provided by the GlycoScience Protocol Online Database (GlycoPOD) (https://jcggdb.jp/GlycoPOD/) of the Japan Consortium for Glycoscience and Glycotechnology (JCGG).

Importantly, the NCBI Glycans hosts the official webpage for the symbol nomenclature for glycans (SNFG) (https://www.ncbi.nlm.nih.gov/glycans/snfg.html) [41, 42], which is the standard graphical representation of glycan structures recommended by the SNFG discussion group (https://www.ncbi.nlm.nih.gov/glycans/snfggroup.html). The SNFG page provides a detailed description of the SNFG standard, along with a list of software tools supporting it. A recent addition to the NCBI Glycans is the webpage for the structural representation of sialic acids (Sia) and other nonulosonic acids (NulOs) using the SNFG (https://www.ncbi.nlm.nih.gov/glycans/sialic.html) [43]. For monosaccharide residues listed on the two web pages, links to corresponding records in PubChem are available, helping users to find additional information about them.

Through the NCBI Glycans, users can readily identify glycan-related records in multiple NCBI resources, including PubMed, PubChem, Structure, Gene, Protein, MedGen, and Bookshelf. The NCBI Glycans also provides the link to some external resources useful for glycoscience research, including the Carbohydrate Structure Databases [16], the carbohydrate-active enzyme (CAZy) database [17], ExPASy Glycomics Resource Page [27], GLYCAM (https://glycam.org/), GlyCosmos Portal [28], GlyGen [26], GlySpace Alliance [29, 30], and GlyTouCan [21, 22].

### Glycans in PubChem Substance and Compound

Launched in 2004, PubChem has been a key chemical information resource for scientific communities as well as the general public. While PubChem primarily contains small molecules, it also has many glycan and glycan-containing chemical structure records submitted by various sources. Using the SDF files of compound records in PubChem (from the PubChem FTP site) as inputs, the Sugar & Splice toolkit from NextMove Software (https://www.nextmovesoftware.com/sugarnsplice.html) detected 1,167,038 compound records containing glycan monomers (Additional File 1). For 142,974 compound records, the PubChem definition of a “biologic” was met, meaning that the structure contains recognized biopolymers, the substituents are known, and Sugar & Splice was able to generate a biologic image or to compute at least one of the following biologic properties (LINUCS [44], IUPAC [45], and IUPAC-condensed [45]) (Additional File 2).

Glycan data submission from GlyTouCan and SNFG to PubChem, which started back in 2015, is an important addition to the existing glycan-related data content. As of June 13, 2024, PubChem contains about 220 thousand glycan substances from these two sources. Most of these substances were submitted by GlyTouCan, with 225 substances from SNFG. Among the ~220 thousand glycan substances, ~40% of them are mapped to 84 thousand compound records in PubChem. The Summary page of each of these compounds presents all information available for that compound in PubChem, including annotations collected from authoritative data sources as well as experimentally determined bioactivity data archived in PubChem. Most of the glycan-specific data available within the Compound Summary are shown in the Biologic Description section, as in the following example (for CID 91846437):

- https://pubchem.ncbi.nlm.nih.gov/compound/91846437#section=Biologic-Description

**Fig. 1:**
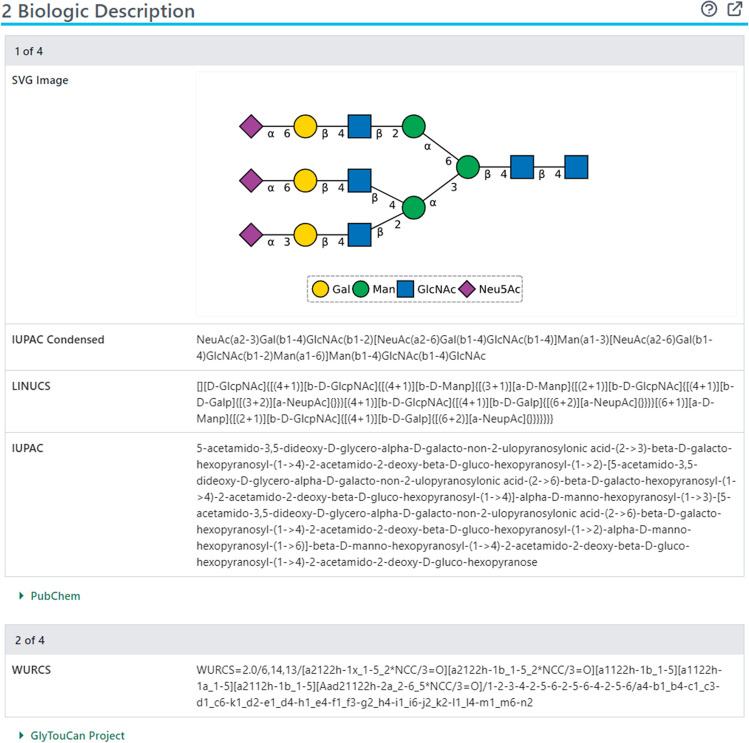
The Biologic Description section of the Compound Summary page of CID 91846437 (https://pubchem.ncbi.nlm.nih.gov/compound/91846437#section=Biologic-Description)

This section (Fig. 1) presents the Scalable Vector Graphics (SVG) image and line notations (LINUCS [44], IUPAC [45], and IUPAC-condensed [45]) of the glycan structure, computed by PubChem using NextMove Software’s Sugar & Splice toolkit. The section also shows various kinds of annotations (e.g., WURCS, classification, monosaccharide composition, motif, permethylated and monoisotopic masses), collected from GlyTouCan, GlyGen, and GlyCosmos (see Table 1).

There are glycan-specific annotations that appear in sections other than the Biologic Description. Examples are the GlyTouCan accession (under the Names and Identifiers section) and the Taxonomy information (in the Taxonomy section), which are accessible via the URLs (when using the same example of CID 91846437):

- https://pubchem.ncbi.nlm.nih.gov/compound/91846437#section=GlyTouCan-Accession
- https://pubchem.ncbi.nlm.nih.gov/compound/91846437#section=Taxonomy

**Fig. 2:**
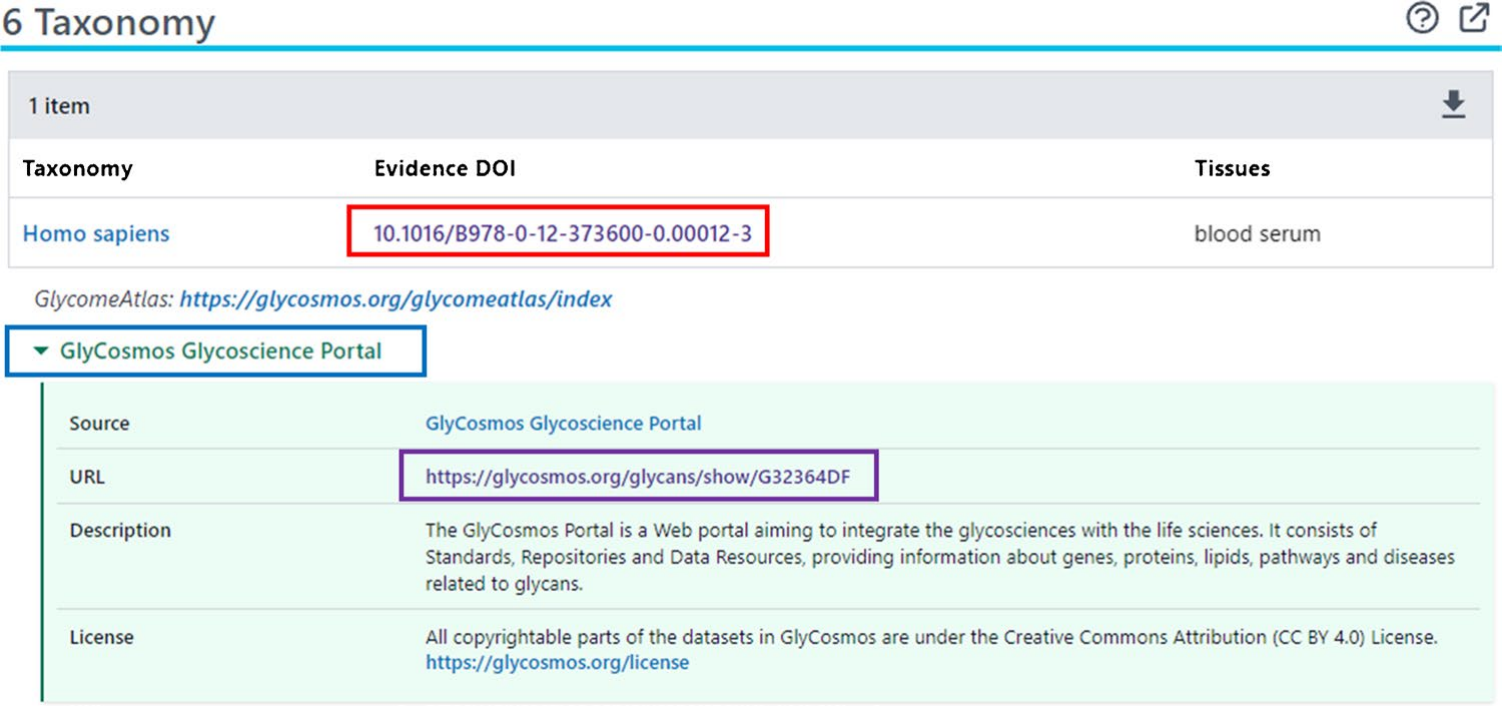
The Taxonomy section of the Compound Summary page of CID 91846437 (https://pubchem.ncbi.nlm.nih.gov/compound/91846437#section=Taxonomy). Clicking the digital object identifier (DOI) (red box) directs to the reference where the taxonomy information was extracted from. Clicking the data source name (blue box) shows metadata for the taxonomy information, along with the link to the corresponding record in the data source (purple box)

It is noteworthy that the Taxonomy section provides information on the organism and tissue where the glycan can be found, which is also presented as “GlyCosmos Species” and “GlyCosmos Tissue/Organ” in the Biologic Description section. However, the Taxonomy section presents that information with the reference where it was extracted from, helping users cross-check the accuracy of the information (see Fig. 2). In addition, all annotations provided on the Summary page of a PubChem record are presented with their provenance information (e.g., which data submitter provided the content), as shown in the blue box in Fig. 2. Clicking the source name shows additional metadata for the annotation, along with the link to the corresponding record in the data source (the purple box in Fig. 2).

### Glycoinformation in PubChem Protein and Gene

The Protein and Gene Summary pages show all available data in PubChem for a given gene or protein. For example, the following URLs are for the Summary pages for human β-1,4-galactosyltransferase 1 (B4GALT1) (NCBI Protein accession P15291) and its encoding gene (NCBI Gene ID 2683):

- https://pubchem.ncbi.nlm.nih.gov/protein/P15291
- https://pubchem.ncbi.nlm.nih.gov/gene/2683

**Fig. 3:**
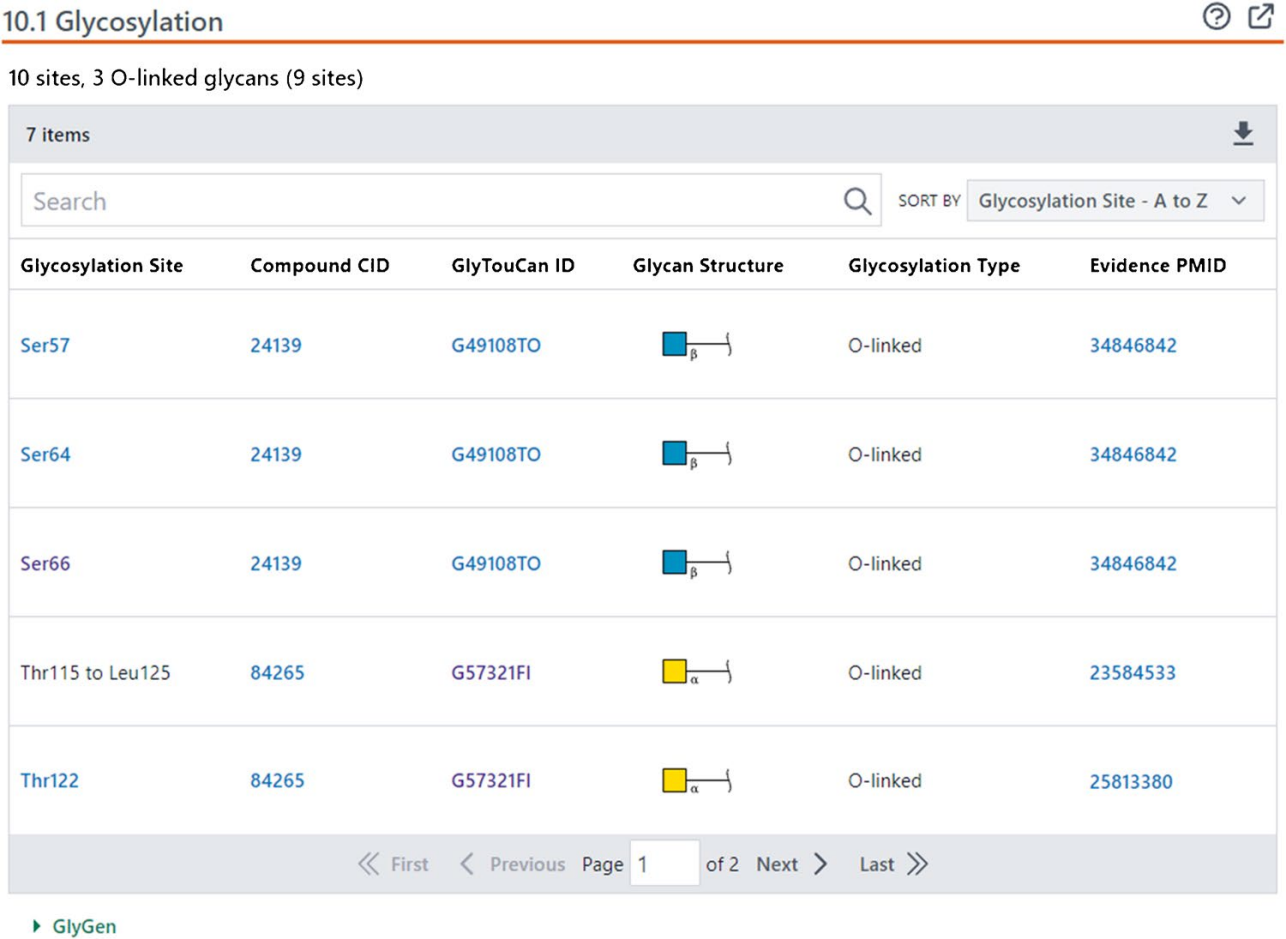
The Glycosylation section of the Protein Summary page of human β-1,4-galactosyltransferase 1 (B4GALT1) (NCBI Protein accession P15291) (https://pubchem.ncbi.nlm.nih.gov/protein/P15291#section=Glycosylation). For each glycosylation site, clicking the Compound CID and GlyTouCan ID directs to the corresponding glycan record in PubChem Compound and GlyTouCan. Clicking the Evidence PMID directs to the PubMed record for the paper where the glycosylation information was reported

These pages contain a wide range of information on the protein and gene, including glycan-related annotations from GlyGen and GlyCosmos (see Table 1). The Protein Summary shows the glycosylation information (e.g., the site and type of glycosylation and the article from which the glycosylation data was extracted) (see Fig. 3), as shown in the following example for the B4GALT1 protein.

- https://pubchem.ncbi.nlm.nih.gov/protein/P15291#section=Glycosylation

In the Gene Summary page, the gene-chemical and gene-disease interaction information, collected from GlyCosmos, is presented with interaction data from other sources (e.g., Comparative Toxicogenomics Database (CTD) [46] and DrugBank [47]), as shown in these examples:

- https://pubchem.ncbi.nlm.nih.gov/gene/2683#section=Chemical-Gene-Interactions
- https://pubchem.ncbi.nlm.nih.gov/gene/2683#section=Gene-Disease-Associations

Importantly, the Protein and Gene Summary pages provide quick access to bioactivity data of chemicals tested against the corresponding protein and gene targets, respectively. As an example, the following URLs lead to the bioactivity data for human B4GALT1 protein and its encoding gene:

- https://pubchem.ncbi.nlm.nih.gov/protein/P15291#section=Tested-Compounds
- https://pubchem.ncbi.nlm.nih.gov/gene/2683#section=Tested-Compounds

These bioactivity data can be downloaded and used to perform a structure-activity relationship analysis or to build a predictive model for bioactivity against the target protein or gene.

### Glycoinformation in PubChem Pathway

The Summary page for a given pathway lists chemicals, proteins, and genes involved in or associated with that pathway, along with information on interactions or reactions among them. As an example, the following URL directs users to the Pathway Summary page for the lysosomal oligosaccharide catabolism pathway (Reactome ID R-HAS-8853383).

- https://pubchem.ncbi.nlm.nih.gov/pathway/Reactome:R-HSA-8853383

The Pathway Summary also shows a list of glycans, glycoproteins, and glycogenes (when available), integrated from GlyCosmos, as shown in these examples:

- https://pubchem.ncbi.nlm.nih.gov/pathway/Reactome:R-HSA-8853383#section=Glycans
- https://pubchem.ncbi.nlm.nih.gov/pathway/Reactome:R-HSA-8853383#section=GlycoProteins
- https://pubchem.ncbi.nlm.nih.gov/pathway/Reactome:R-HSA-8853383#section=GlycoGenes

### Glycoinformation in PubChem Taxonomy

The Taxonomy Summary page for a taxon can be accessed through a URL containing the corresponding NCBI Taxonomy ID. For example, the Taxonomy Summary page for Danio rerio (zebrafish; NCBI Taxonomy ID 7955) can be accessed via the URL:

- https://pubchem.ncbi.nlm.nih.gov/taxonomy/7955

Among the wide range of information presented in the Taxonomy Summary is the list of glycans found in the organism, the tissue or organ where the glycans were found, and the links to the articles where the information was extracted from. For example, the following URL leads to the list of glycans associated with zebrafish.

- https://pubchem.ncbi.nlm.nih.gov/taxonomy/7955#section=Glycans

In addition, the Taxonomy Summary page shows the whole-organism bioassays performed on the whole taxon without a specific target gene or protein as well as the bioactivity data for the chemicals tested in those assays, as in the following examples:

- Whole-organism bioassays for zebrafish

https://pubchem.ncbi.nlm.nih.gov/taxonomy/7955#section=Whole-Organism-BioAssays

- Whole-organism bioactivities for zebrafish

https://pubchem.ncbi.nlm.nih.gov/taxonomy/7955#section=Whole-Organism-Bioactivities

### Searching for glycan-related data in PubChem

**Fig. 4:**
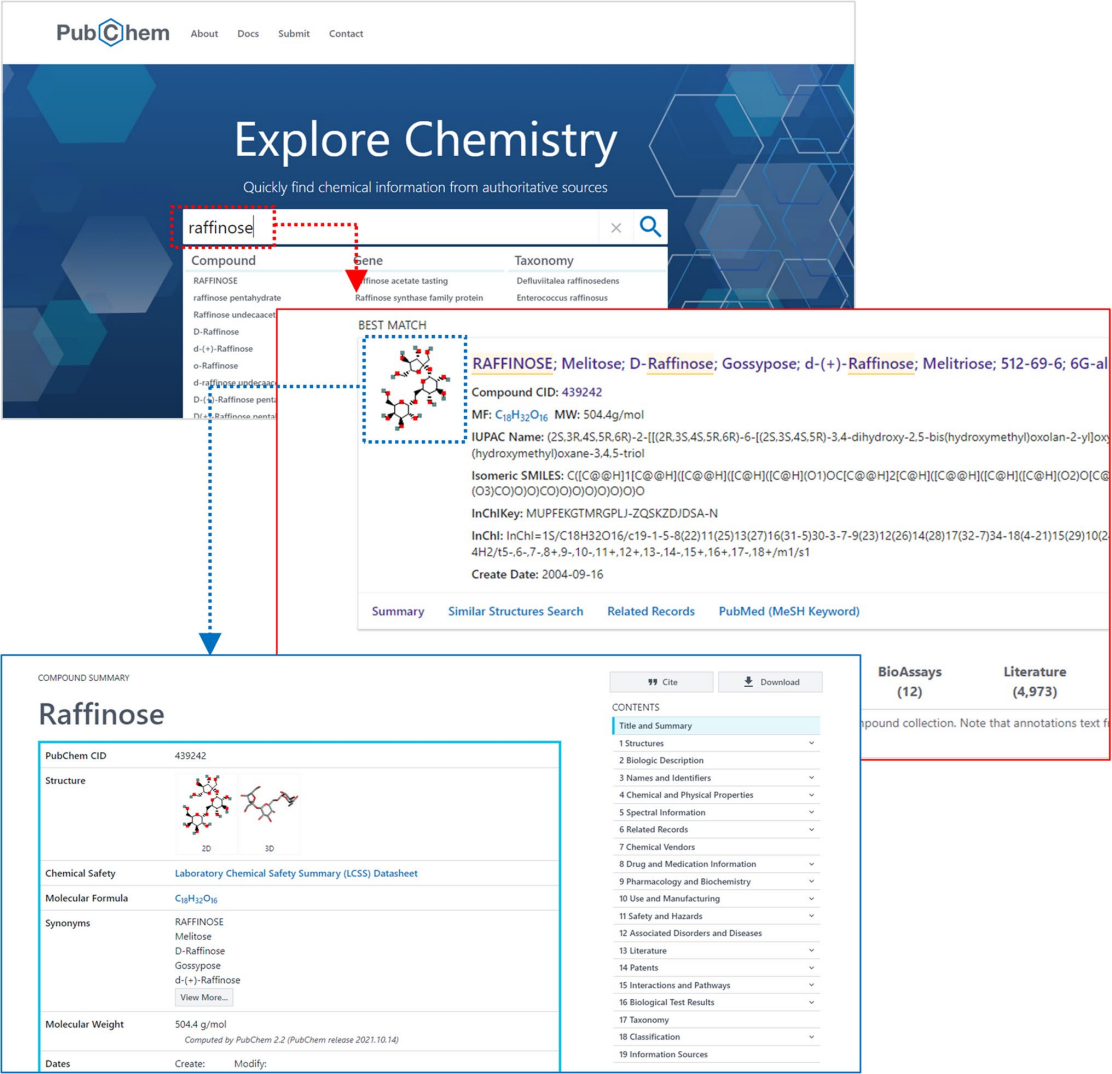
Searching PubChem for “raffinose” (a trisaccharide commonly found in plants). When a keyword query is provided, the search result page presents records returned from the search, with the most relevant record at the top. Clicking one of the hit records directs to the Summary page for that record. See References [32, 33] for various tasks that can be done on the search result page

Users can initiate a search of PubChem by providing a keyword query in the search box available on the PubChem home page (Fig. 4). PubChem accepts various types of keyword queries, including chemical names, chemical abstract service (CAS) registry numbers, PubChem record identifiers (SID, CID, and AID), gene/protein names and symbols, and disease names. To search for glycans and glycan-related records, a GlyTouCan Accession may also be used as a query.

When a keyword query is provided, PubChem searches all data collections simultaneously and returns a list of hit records for each collection. PubChem also tries to identify the most relevant record and presents it at the top of the search result page. Clicking one of the hit records will direct the user to its Summary page. In addition, the search result page allows users to perform various tasks, such as downloading the hit records, filtering the hit list based on certain attributes, and combining hit lists from multiple queries. More detailed information on these tasks can be found in our previous papers [32, 33].

### Getting glycan-related annotations from a source

**Fig. 5:**
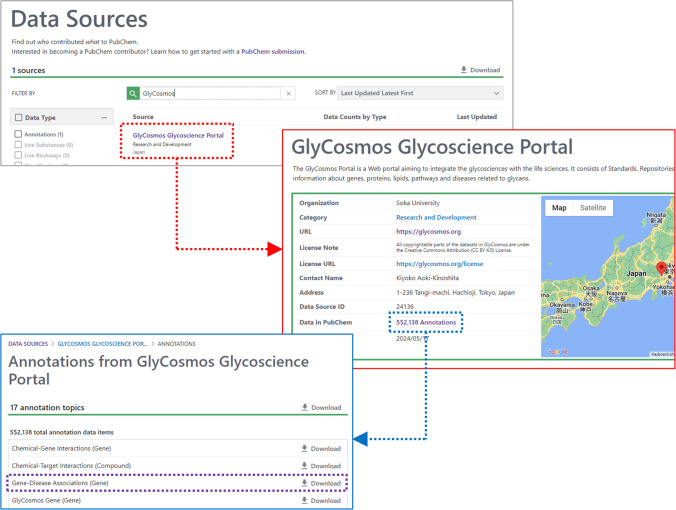
Getting all gene-disease associations from the GlyCosmos Portal in PubChem. The PubChem Data Sources page (https://pubchem.ncbi.nlm.nih.gov/source/) allows users to search for a data source of interest and move to the dedicated web page for that source, where annotations from the source can be downloaded on a local machine

PubChem users are often interested in getting all available annotations from a particular source, for example, getting all gene-disease associations from the GlyCosmos Glycoscience Portal. It can be readily done using the PubChem Data Sources page (https://pubchem.ncbi.nlm.nih.gov/source/) (see Fig. 5), which lists all PubChem data sources. This page allows users to search the sources by name and filter them based on the source category, status, and country as well as the type of data from the source. Clicking one of the sources in this list directs to a dedicated web page for that source, which provides information on the source as well as the link to the data originating from the source. Figure 5 shows how to download the gene-disease associations from the GlyCosmos Portal.

### Programmatic access

Annotations to PubChem records can be programmatically accessed via PUG-View (https://pubchem.ncbi.nlm.nih.gov/docs/pug-view) [48], which is one of the REST-ful interfaces provided by PubChem. For example, the following PUG-View request URL downloads the annotations presented in the Biologic Description section of the Compound Summary page for CID 91846437.

- https://pubchem.ncbi.nlm.nih.gov/rest/pug_view/data/compound/91846437/JSON?heading=Biologic+Description

It is also possible to download a particular kind of annotation for all records from a given source. For example, all glycan classification annotations from GlyGen can be downloaded via the following URL:

- https://pubchem.ncbi.nlm.nih.gov/rest/pug_view/annotations/heading/JSON/?source=GlyGen&heading_type=Compound&heading=GlyGen%20Classification&page=1&response_type=display

It is noteworthy that a PUG-View request returns up to 1000 annotations, although some headings have more annotations than this limit. Therefore, data from PUG-View requests are paginated. At the end of the returned output, the “TotalPages” and “Page” values are included to indicate the total pages of the data and the page number for the returned data. If the “TotalPages” value is greater than 1, there is more data to download. By default, the “Page” value is set to 1, meaning that the first page of the data is returned. To get another page of the data, the “Page” value should be adjusted accordingly. For example, the following request URL retrieves the second page of the glycan classification annotations from GlyGen:

- https://pubchem.ncbi.nlm.nih.gov/rest/pug_view/annotations/heading/JSON/?source=GlyGen&heading_type=Compound&heading=GlyGen%20Classification&page=2&response_type=display

### Accessing related glycans with different subsumption levels

**Fig. 6:**
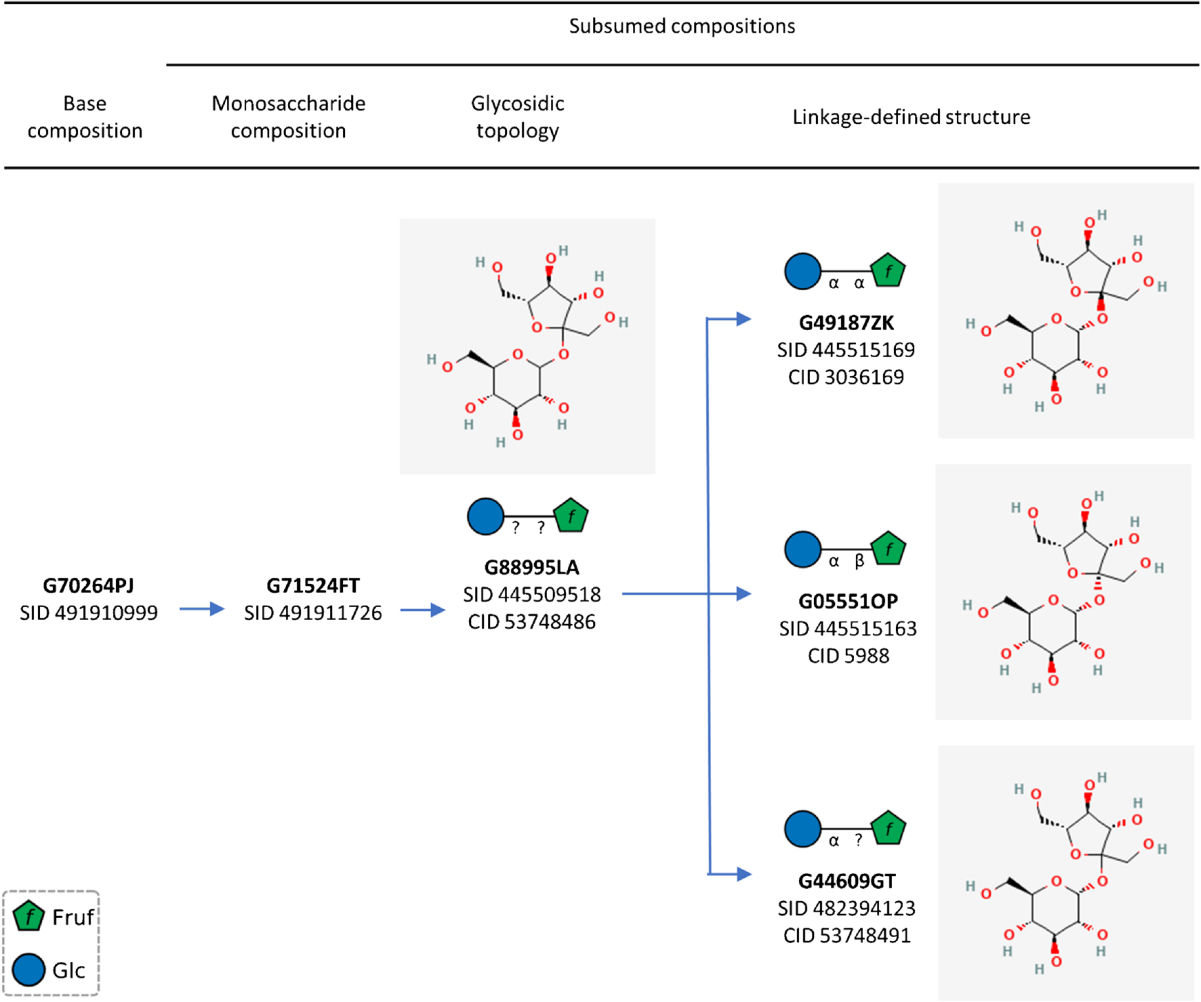
Sucrose (CID 5988; GlyTouCan ID: G05551OP) and related glycans with different subsumption levels. Note that glycans with no glycosidic linkage information do not have corresponding compound records in PubChem (i.e., no CIDs)

The subsumption of a glycan record refers to the level of the structural details known for the glycan (https://glycosmos.org/help). There are five subsumption levels: base composition, monosaccharide composition, glycosidic topology, linkage-defined saccharide, and fully defined saccharide (in the order of increasing structural details) (see Table 2). Figure 6 shows how glycans with different subsumption levels are integrated into PubChem. It is noteworthy that some glycans have no CIDs (i.e., no corresponding records in the Compound collection). While all compound records in PubChem are required to have discrete chemical structures, glycans whose subsumption level is “base composition” or “monosaccharide composition” (e.g., G70264PJ and G71524FT in Fig. 6) do not have sufficient information on the glycosidic linkage (i.e., anomeric configuration and the carbon numbers of the monosaccharides which are linked together). Therefore, they cannot be added to PubChem Compound. On the other hand, the glycans in Fig. 6 whose subsumption level is “glycosidic topology” or “linkage-defined saccharide” have CIDs in PubChem and that they have the same atom connectivity with varying stereochemistry for the glycosidic linkage. In general, only glycans whose subsumption level is “glycosidic topology” or higher can have discrete structures and hence corresponding records in PubChem Compound.

**Table 2:** Definition of glycan subsumption levels

Subsumption level	Definition	Can have a CID?
Fully defined saccharide	All glycosidic linkage information (including anomeric configuration and the carbon numbers of the monosaccharides which are linked together) is specified.	Yes
Linkage-defined saccharide	Information on *at least* one glycosidic linkage information is specified.	Yes
Glycosidic topology	Information on the monosaccharides that are linked together is identified, but linkage conformations are all unknown.	Maybe
Monosaccharide composition	The number of monosaccharides (with known stereospecificity, such as galactose, or N-acetylglucosamine) is known but no linkages are specified.	No
Base composition	The number of base types (such as hexose, deoxyhexose) and no linkages are specified.	No

The Glycan Naming and Subsumption Ontology (GNOme) [49] is added to the PubChem Classification Browser, which allows users to retrieve PubChem records that belong to a certain class or have particular annotations.

- https://pubchem.ncbi.nlm.nih.gov/classification/#hid=133

After selecting “GNOme” from the “Select classification” dropdown menu, users can navigate the GNOme classification tree to identify the node corresponding to a glycan or glycans with a desired monoisotopic mass value or range. This enables users to retrieve the glycans with the same mass but with different subsumption levels.

### Limitations and future directions

As mentioned previously, only 40% of the 220 thousand glycan substance records from GlyTouCan are associated with compound records. The remaining 60% do not have associated compound records, primarily due to substances having a variable (non-discrete or incomplete) aspect. Some glycans do not have sufficient information on glycosidic linkages, yet Compound records do not allow such variability, and therefore, no corresponding compound CID records in PubChem can be associated. However, because these glycans may still have some useful information, it is necessary to provide a way to allow users to access information on them. Therefore, a dedicated web page is being considered for each of the glycans with non-discrete structures. A brief explanation of recent additions of Compound summary records for non-discrete chemical substances in PubChem is given in our recent publication [50].

Many chemical structures in the PubChem Compound database contain glycans. As of June 13, 2024, a total of 1,167,038 records in the PubChem Compound database contain a glycan monomer as determined by the Sugar & Splice software from NextMove Software (Additional File 1). However, there is no definitive definition of “what is a glycan monomer?” Many of the unique glycan monomers within PubChem are synthetically created or synthetically modified from natural glycans. Of these 1.1 M glycan-containing CIDs in PubChem Compound, only 142,974 could have a computed image and/or other computed properties using Sugar & Splice (Additional File 2), which is to say that they more closely resemble a glycan structure than a glycoconjugate or some other glycosylated chemical structure. This suggests that most of the 1.1 M glycan-containing structures in PubChem are part of a larger chemical structure (e.g., a glycoconjugate). The absence of a well-defined definition of “what is a glycan?” or “what is a naturally occurring glycan?” makes it difficult to assess the portion of PubChem that may be of interest to glycan researchers. It also highlights opportunities to assess parts of PubChem (or other chemical database resources) that contain useful glycan-related information that may assist researchers in making new discoveries. It also highlights opportunities for data exchange between chemical databases and glycan-focused data systems. While most information has flowed from glycan-focused resources to chemical databases, it is possible for this exchange to work in the reverse direction, once the scope of the glycan-containing structures can be assessed (e.g., which chemical structures are potentially of importance to researchers working with glycans).

## Conclusions

The present paper describes two NCBI resources, the NCBI Glycans and PubChem, which provide glycan-related information useful for the glycoscience research community. The NCBI Glycans provides quick access to various glycoscience data contents, including the two online books, the “Essentials of Glycobiology” and “Glycoscience Protocols,” and the SNFG official web page. It also allows users to identify glycan-related records in NCBI databases, such as PubMed, PubChem, Structure, Gene, Protein, MedGen, and Bookshelf. In addition, the NCBI Glycans provides links to popular glycoscience information resources external to NCBI.

As a large-scale public chemical database, PubChem contains a substantial number of glycans and related data, integrated from important glycoscience resources like GlyTouCan, GlyCosmos, GlyGen, and GlycoNAVI. The glycan-related data in PubChem are organized into multiple data collections (i.e., Substance, Compound, Protein, Gene, Pathway, and Taxonomy) and presented on the Summary page of relevant PubChem records. These records can be searched for using a keyword query (e.g., names or identifiers). The PubChem Classification Browser and Data Sources page are also useful tools to access glycan data in PubChem. It is also possible to programmatically access the glycan data using the PUG-View programmatic service. In the spirit of open data exchange, for all glycan data integrated from external data sources, PubChem provides links back to the corresponding record at the original source’s website (see Fig. 2). This allows users to check the accuracy of the data and find additional information at the data source.

The PubChem team has been actively working with the glycoscience community. In collaboration with the SNFG discussion group, it maintains the SNFG guideline page and the sialic acid nomenclature pages at the NCBI Glycans. As explained in our recent paper [51, 52], the PubChem team collaborates with the developers of other glycoscience databases, such as GlyTouCan, GlyGen, and GlyCosmos, to make glycan-related data comply with the FAIR guiding principle for scientific data management and stewardship [53], where FAIR is an acronym for “findable, accessible, interoperable, and reusable.” These efforts will improve the accessibility of glycan-related information, enabling scientists to exploit them in their research.

## Supplementary Information

Below is the link to the electronic supplementary material.

Supplementary File 1 A list of CIDs of the 1,167,038 compounds whose structure contains a glycan monomer unit, detected by the Sugar & Splice toolkit from NextMove Software (GZ 2.77 MB)

Supplementary File 2 A list of CIDs of the 142,974 compounds for which the Sugar & Splice toolkit could generate a biologic image or compute at least one of the biologic properties (LINUCS, IUPAC, and IUPAC-condensed) (GZ 3.14 MB)

## Data Availability

All PubChem data are freely available to the public.
